# Supplementary material for: The ascending arousal system shapes neural dynamics to mediate awareness of cognitive states
Source: Nat Commun. 2021 Oct 14;12:6016. doi: 10.1038/s41467-021-26268-x (PMC8516926; doi:10.1038/s41467-021-26268-x)
Supplement: Supplementary file 1 — Supplementary information. [file 41467_2021_26268_MOESM1_ESM.pdf]

Supplementary Figures

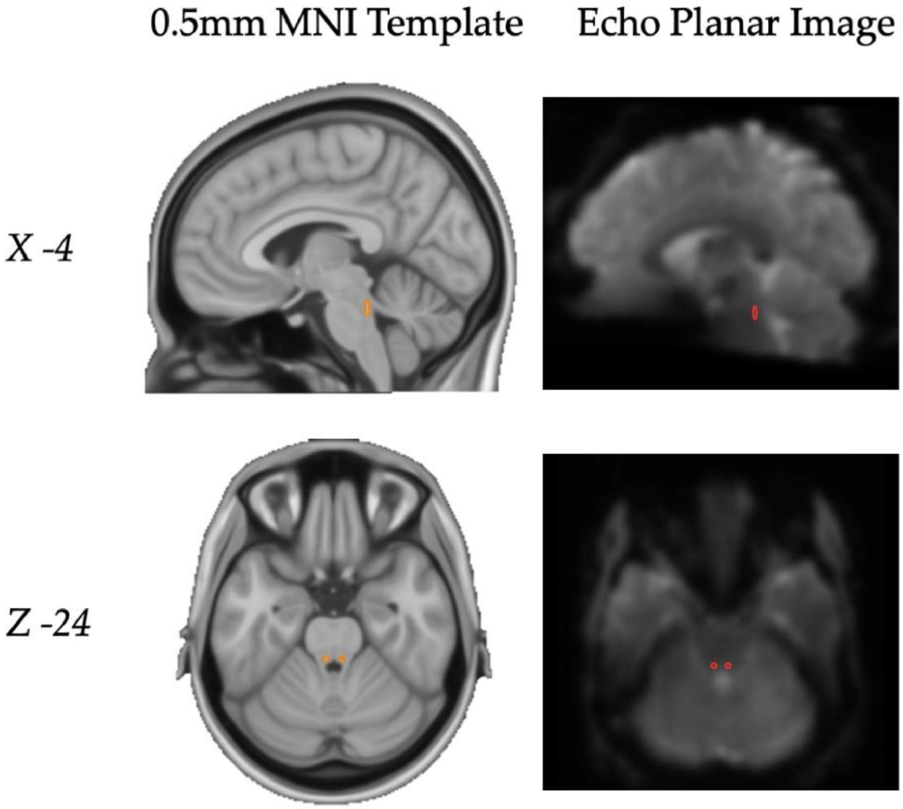

Supplementary Fig. 1. The locus coeruleus. Left: The anatomical locus coeruleus mask projected onto MNI 0.5mm standard brain (orange); Right: The anatomical locus coeruleus mask down-sampled onto an example 7T Echo Planar Image from a single subject (red).

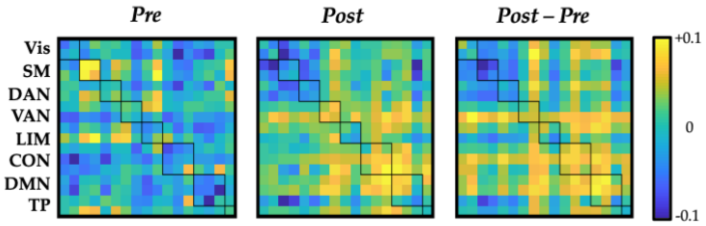

Supplementary Fig. 2. Time-varying correlations. Average correlation preceding (left) and following (middle) the zero-lagged τ_{LC-BNM} value, along with the difference between the two (right); squares represent eight pre-defined sub-networks: Vis – visual, SM – somatomotor, DAN – dorsal attention, VAN – ventral attention, LIM – limbic, CON – control, DMN – default and TP – temporal pole.

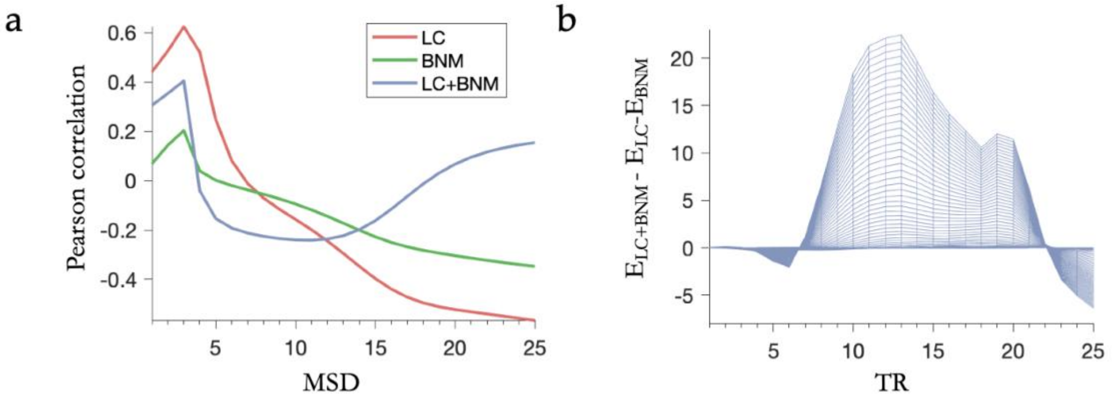

Supplementary Fig. 3 – Simultaneous LC and BNM phasic bursts move dynamics to differing regimes. **a** Pearson correlation of the energy profile with the HRF at different MSD. **b** The energy landscape of simultaneous LC+BNM phasic bursts relative to their linear superposition, suggesting the simultaneous combination may allow the system to reach particularly unique brain-states that neither individually could reach.
