# Supplementary material for: The ascending arousal system shapes neural dynamics to mediate awareness of cognitive states
Source: Nat Commun. 2021 Oct 14;12:6016. doi: 10.1038/s41467-021-26268-x (PMC8516926; doi:10.1038/s41467-021-26268-x)
Supplement: Supplementary file 3 — Reporting summary. [file 41467_2021_26268_MOESM3_ESM.pdf]

nature research

Corresponding author(s): Brandon Munn

Last updated by author(s): Jul 21, 2021

Reporting Summary

Nature Research wishes to improve the reproducibility of the work that we publish. This form provides structure for consistency and transparency in reporting. For further information on Nature Research policies, see our Editorial Policies and the Editorial Policy Checklist.

Statistics

For all statistical analyses, confirm that the following items are present in the figure legend, table legend, main text, or Methods section.

n/a	Confirmed	
☐	☒	The exact sample size (*n*) for each experimental group/condition, given as a discrete number and unit of measurement
☐	☒	A statement on whether measurements were taken from distinct samples or whether the same sample was measured repeatedly
☐	☒	The statistical test(s) used AND whether they are one- or two-sided<br>*Only common tests should be described solely by name; describe more complex techniques in the Methods section.*
☐	☒	A description of all covariates tested
☐	☒	A description of any assumptions or corrections, such as tests of normality and adjustment for multiple comparisons
☐	☒	A full description of the statistical parameters including central tendency (e.g. means) or other basic estimates (e.g. regression coefficient) AND variation (e.g. standard deviation) or associated estimates of uncertainty (e.g. confidence intervals)
☐	☒	For null hypothesis testing, the test statistic (e.g. *F*, *t*, *r*) with confidence intervals, effect sizes, degrees of freedom and *P* value noted<br>*Give P values as exact values whenever suitable.*
☒	☐	For Bayesian analysis, information on the choice of priors and Markov chain Monte Carlo settings
☒	☐	For hierarchical and complex designs, identification of the appropriate level for tests and full reporting of outcomes
☐	☒	Estimates of effect sizes (e.g. Cohen's *d*, Pearson's *r*), indicating how they were calculated

Our web collection on statistics for biologists contains articles on many of the points above.

Software and code

Policy information about availability of computer code

Data collection

At the Centre for Advanced Imaging, The University of Queensland, imaging data were collected using a 7 T Siemens MR scanner fitted with a 32-channel head coil. For resting-state fMRI, whole-brain echo-planar images were acquired using a multiband sequence (acceleration factor, 5; Moeller et al., 2010). 1050 volumes were collected (?10 min each) with the following parameters: voxel size, 2 mm3; TR = 586 ms; TE = 23 ms; flip angle, 40°; FOV, 208 mm; 55 slices. Structural images were also collected to assist functional data preprocessing. These images were acquired using the following parameters: MP2RAGE sequence; voxel size, 0.75 mm3; TR = 4300 ms; TE = 3.44 ms; 256 slices.

Imaging data were preprocessed using an adapted version of Matlab (MathWorks R2020a, RRID: SCR_001622) toolbox Data Processing Assistant for Resting-State fMRI (DPARSF V 3.0; Chao-Gan and Yu-Feng, 2010; RRID: SCR_002372). DICOM (Digital Images and Communications in Medicine) images were first converted to Nifti format and realigned. T1 images were reoriented, skull-stripped [FMRIB (Oxford Centre of Functional MRI of the Brain) Software Library Brain Extraction Tool (FSL BET); RRID: SCR_009472], and coregistered to the Nifti functional images using statistical parametric mapping (SPM8; RRID: SCR_007037) functions. Segmentation and the DARTEL (diffeomorphic anatomical registration through exponentiated lie algebra) algorithm were used to improve the estimation of non-neural signal in subject space and the spatial normalization (Ashburner, 2007). From each gray matter voxel, the following signals were regressed: undesired linear trends, signals from the six head-motion parameters (three translation, three rotation), white matter, and CSF (estimated from single-subject masks of white matter and CSF). The CompCor method (Behzadi et al., 2007) was used to regress out residual signal unrelated to neural activity (i.e., five principal components derived from noise regions-of-interest in which the time series data were unlikely to be modulated by neural activity). We employed a strict head-motion censoring approach (see below). Single-subject functional images were subsequently normalized and smoothed using DARTEL (4 mm3). Data processing steps also involved filtering (0.01–0.15 Hz) at a low-frequency component of the BOLD signal known to be sensitive to resting-state functional connectivity (Sun et al., 2004).

The Louvain modularity algorithm from the Brain Connectivity Toolbox (BCT; http://www.brain-connectivity-toolbox.net/) was used on the neural network edge weights to estimate community structure.

Data analysis	Available on github.com/bmunn/BSI

For manuscripts utilizing custom algorithms or software that are central to the research but not yet described in published literature, software must be made available to editors and reviewers. We strongly encourage code deposition in a community repository (e.g. GitHub). See the Nature Research guidelines for submitting code & software for further information.

Data

Policy information about availability of data

All manuscripts must include a data availability statement. This statement should provide the following information, where applicable:

- Accession codes, unique identifiers, or web links for publicly available datasets
- A list of figures that have associated raw data
- A description of any restrictions on data availability

The resting-state BOLD cortical activity, subcortical LC and BNM activity, network statistics, and LC and BNM masks in MNI space have been deposited in a Zenodo database (https://doi.org/10.5281/zenodo.5315132). The raw resting-state BOLD data that support the findings of this study were obtained from (Hearne et al., 2017) and they are available from (http://data.qld.edu.au/public/Q1361/). The raw meditation dataset was obtained from (Hasenkamp et al., 2012), access can be obtained from the authors upon reasonable request.

Field-specific reporting

Please select the one below that is the best fit for your research. If you are not sure, read the appropriate sections before making your selection.

☒ Life sciences ☐ Behavioural & social sciences ☐ Ecological, evolutionary & environmental sciences

For a reference copy of the document with all sections, see nature.com/documents/nr-reporting-summary-flat.pdf

Life sciences study design

All studies must disclose on these points even when the disclosure is negative.

Sample size	We did not collect the data. The resting-state data had a sample size of n= 65. The meditation dataset had a sample size of n = 14.
Data exclusions	6 individuals were excluded from the resting-state date leaving n= 59 in the final analysis (four participants were excluded due to MR scanning issues, one participant was excluded due to an unforeseen brain structure abnormality, and one was excluded due to inconsistent BOLD dynamics following global-signal regression).<br>All individuals were analysed from the meditation dataset.
Replication	Resting state results were replicated in HCP data.
Randomization	Individuals were not grouped.
Blinding	All data was analysed blind to individuals, individuals were not grouped.

Reporting for specific materials, systems and methods

We require information from authors about some types of materials, experimental systems and methods used in many studies. Here, indicate whether each material, system or method listed is relevant to your study. If you are not sure if a list item applies to your research, read the appropriate section before selecting a response.

Materials & experimental systems

n/a	Involved in the study	
☒	☐	Antibodies
☒	☐	Eukaryotic cell lines
☒	☐	Palaeontology and archaeology
☒	☐	Animals and other organisms
☐	☒	Human research participants
☒	☐	Clinical data
☒	☐	Dual use research of concern

Methods

n/a	Involved in the study	
☒	☐	ChIP-seq
☒	☐	Flow cytometry
☒	☐	MRI-based neuroimaging

Human research participants

Policy information about studies involving human research participants

Population characteristics	In the resting-state data sixty-five healthy, right-handed adult participants (mean, 23.35 years; SD, 3.6 years; range 18–33

Population characteristics	years) were recruited.<br>In the meditation dataset fourteen healthy right-handed adult meditation practitioners, ages 28–66 (3 male) were recruited.
Recruitment	Resting-state dataset: Individuals were recruited from the Brisbane area and were required to be right-handed.<br>Meditation dataset: Individuals were recruited from local Atlanta meditation communities and were required to practice mediation regularly for over a year. They were excluded for fMRI contraindications, current substance dependence, history of sustained loss of consciousness, major neurological or medical illness, left-handedness, pregnancy, or history of major mental illness (as assessed by the Structured Clinical Interview for DSM-IV, Axis-I, Non-Patient version; First et al., 2001).
Ethics oversight	Resting-state dataset: Participants provided informed written consent to participate in the study. The research was approved by The University of Queensland Human Research Ethics Committee.<br>Meditation dataset: All participants signed a consent form approved by the Institutional Review Board at Emory University and the Atlanta Veterans Affairs Research and Development Committee as an indication of informed consent.

Note that full information on the approval of the study protocol must also be provided in the manuscript.
